# Classical Risk Factors and Inflammatory Biomarkers: One of the Missing Biological Links between Cardiovascular Disease and Major Depressive Disorder

**DOI:** 10.3390/ijms19061740

**Published:** 2018-06-12

**Authors:** Thomas C. Baghai, Gabriella Varallo-Bedarida, Christoph Born, Sibylle Häfner, Cornelius Schüle, Daniela Eser, Peter Zill, André Manook, Johannes Weigl, Somayeh Jooyandeh, Caroline Nothdurfter, Clemens von Schacky, Brigitta Bondy, Rainer Rupprecht

**Affiliations:** 1 Department of Psychiatry and Psychotherapy, Ludwig-Maximilian-University of Munich, Nußbaumstraße 7, D-80336 Munich, Germany; Christoph.Born@vivantes.de (C.B.); sibylle.haefner@med.uni-heidelberg.de (S.H.); Cornelius.Schuele@med.uni-muenchen.de (C.S.); Daniela.Eser@med.uni-muenchen.de (D.E.); Peter.Zill@med.uni-muenchen.de (P.Z.); brigitta.bondy@med.uni-muenchen.de (B.B.); Rainer.Rupprecht@medbo.de (R.R.); 2 Department of Psychiatry and Psychotherapy, University Regensburg, Universitätsstraße 84, D-93053 Regensburg, Germany; Andre.Manook@medbo.de (A.M.); Johannes.Weigl@medbo.de (J.W.); Somayeh.MohammadiJooyandeh@medbo.de (S.J.); Caroline.Nothdurfter@medbo.de (C.N.); 3 Department of Internal Medicine—Preventive Cardiology, Ludwig-Maximilian-University of Munich, Ziemssenstraße 1, D-80336 Munich, Germany; Gabriella.Bedarida@pfizer.com (G.V.-B.); Clemens.vonSchacky@med.uni-muenchen.de (C.v.S.); 4 Max-Planck Fellow at the Max-Planck-Institute for Psychiatry, Kraepelinstraße 2-10, D-80804 Munich, Germany

**Keywords:** cardiovascular disease, cell adhesion molecules, immunology, inflammation, nervous system

## Abstract

Background: Cardiovascular disorders (CVD) and major depressive disorder (MDD) are the most frequent diseases worldwide responsible for premature death and disability. Behavioral and immunological variables influence the pathophysiology of both disorders. We therefore determined frequency and severity of MDD in CVD and studied whether MDD without CVD or other somatic diseases influences classical and inflammatory biomarkers of cardiovascular risk. In addition, we investigated the influence of proinflammatory cytokines on antidepressant treatment outcome. Methods: In a case-control design, 310 adults (MDD patients without CVD, CVD patients, and cardiologically and psychiatrically healthy matched controls) were investigated. MDD patients were recruited after admission in a psychiatric university hospital. Primary outcome criteria were clinical depression ratings (HAM-D scale), vital signs, classical cardiovascular risk factors and inflammatory biomarkers which were compared between MDD patients and healthy controls. Results: We detected an enhanced cardiovascular risk in MDD. Untreated prehypertension and signs directing to a metabolic syndrome were detected in MDD. Significantly higher inflammatory biomarkers such as the high sensitivity C-reaktive protein (hsCRP) and proinflammatory acute phase cytokines interleukine-1β (IL-1β) and interleukine-6 (IL-6) underlined the higher cardiovascular risk in physically healthy MDD patients. Surprisingly, high inflammation markers before treatment were associated with better clinical outcome and faster remission. The rate of MDD in CVD patients was high. Conclusions: Patients suffering from MDD are at specific risk for CVD. Precise detection of cardiovascular risks in MDD beyond classical risk factors is warranted to allow effective prophylaxis and treatment of both conditions. Future studies of prophylactic interventions may help to provide a basis for prophylactic treatment of both MDD and CVD. In addition, the high risk for MDD in CVD patients was confirmed and underlines the requirement for clinical attention.

## 1. Introduction

Cardio- and cerebrovascular disorders are recognized worldwide as the most frequent causes for death and major depressive disorder (MDD) for disability [1]. In recent cross-sectional studies, repeatedly an association of current depressive symptoms and lifetime depressive episodes with cardiovascular disease (CVD), could be confirmed [2]. MDD represents a major risk factor for CVD and for myocardial infarction (MI) [3] independent of traditional risk factors [4], this seems to be especially true in geriatric depression [5]. The MDD subtype, severity of depression [6], and an activation of central stress regulatory systems [7] seem to be relevant in this context. Thereby, the influence of MDD on CVD is equivalent to somatic risk factors [8] and a bi-directional relationship between the cardiovascular system and altered mood states related to an inflammatory status has been suggested [9].

CVD is a chronic inflammatory disease [10] that can be monitored using inflammatory biomarkers such as C-reactive protein (CRP), adhesive cell-surface glycoproteins (sVCAM-1), the monocyte chemotactic protein-1 (MCP-1), the pro-inflammatory acute phase cytokines inteleukin-1 and -6 (IL-1, IL-6), cell adhesion molecules (leukocyte (L-), endothelial (E-), and platelet (P-)selectin), and the intracellular adhesion molecule-1 (sICAM-1) [11], which is acting together with VCAM-1 as monocyte and T cells receptors [11]. Inflammatory mediators and adhesion molecules are peripheral clinical markers for vascular wall inflammation [12] and represent risk factors associated strongly with atherosclerosis [13] and the risk for MI or stroke [14]. They offer prognostic relevance both on the short-run and in the long term in chronic inflammatory states [15].

High CRP seems to reflect also the activity of bipolar disorder: It was associated with high activity of the disorder in both conditions manic and depressed states [16]. Whereas high plasma cholesterol levels are a risk factor in CVD, the use as a clinical marker for affective disorders was a matter of debate and is still unclear [17]. The association of low cholesterol levels with the risk for suicide in psychiatric patients seems to be a more stable finding, but is also discussed controversially up to now [18].

MDD symptoms can be mimicked by excessive secretion of the pro-inflammatory macrophage cytokines [19] and are often accompanied by a hyperactivity of the hypothalamic-pituitary-adrenal (HPA) axis [20] which includes direct stimulatory influence of interleukins (IL-1, IL-6) on hypothalamic corticotropin-releasing factor (CRF) and pituitary corticotropin (ACTH) secretion [19]. Pro-inflammatory cytokines can influence glucocorticoid receptor resistance and serotonergic neurotransmission [21]. Therefore, a causal relationship between inflammation and MDD seems plausible [22,23].

Both population based [23,24,25] and prospective [26] studies implied that MDD belongs to the group of chronic inflammatory diseases, but also controversial results have been obtained in cross-sectional studies which assessed correlations of inflammatory markers and depressive symptoms assessed using self-rating scores [27].

Therefore, the presented comparative prospective case-control study in both diagnostic groups MDD and CVD which directly compares immunological and clinical cardiovascular risk variables may help to clarify these interdependencies. We prospectively investigated both MDD and CVD patients in comparison to psychiatrically and medically healthy controls. We studied frequency and severity of depression in CVD and compared clinical and immunological cardiovascular risk factors between CVD and MDD in an interdisciplinary study. Moreover, we studied to what extent MDD is related to cardiovascular risk factors and to inflammatory biomarkers in cardiovascularly healthy patients suffering from MDD in relation to healthy controls without CVD in order to identify biomarkers which may be suitable for estimation of the cardiovascular risk in MDD. In addition, we investigated the influence of inflammatory markers on antidepressant treatment outcome.

## 2. Results

### 2.1. Classical Cardiovascular Risk Markers

**Figure 1:**
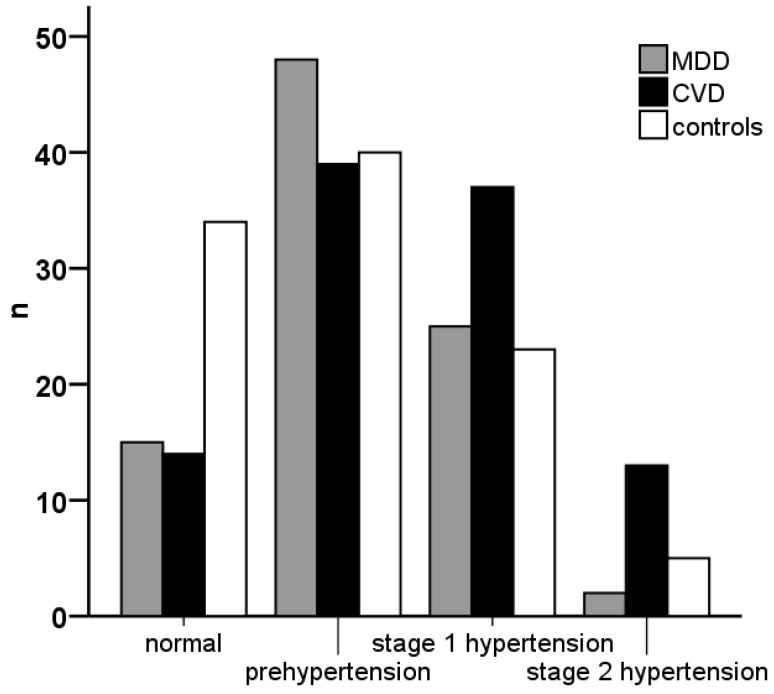
JNC 7 classification of blood pressure. Classification of blood pressure baseline values according “The Seventh Report of the Joint National Committee on Prevention, Detection, Evaluation, and Treatment of High Blood Pressure” (JNC-7) [28] showed significantly different distribution of hypertension categories in major depressive disorder (MDD) and cardiovascular disorder (CVD) patients as well as in healthy controls (n = number of subjects in either group).

Comparison of mean values showed significant differences in a variety of risk markers indicating the elevated cardiovascular risk of MDD patients in comparison to healthy controls (Table 1). Blood pressure (BP) determinations according to the Joint National Committee on Prevention, Detection, Evaluation and Treatment of High Blood Pressure (JNC)-7 definitions [28] (Figure 1) revealed significantly more normal values in healthy controls, prehypertensive BP in MDD and stage 1 and 2 hypertension in CVD patients (χ^2^ = 25.6, d.f. = 6, *p* < 0.001).

Post-hoc comparison of depressed patients and controls confirmed the significantly elevated diastolic blood pressure (BP) (U = 3777, *p* = 0.034) and heart rates (U = 1574, *p* < 0.001). Metabolic risk factors included significantly lower total cholesterol (U = 2622, *p* = 0.042) in view of significantly higher triglycerides (U = 2221, *p* = 0.001) and fasting glucose values (U = 1917, *p* = 0.020) in combination with lower high density lipoproteins (HDL) (U = 1711, *p* = 0.017). Lifetime smoking habits did not differ, whereas more MDD patients are current smokers. After correction for age using exact matching all significant differences except for cholesterol (*p* = 0.24) were confirmed. Moreover, systolic BP was significantly higher in MDD patients at baseline (U = 2396, *p* = 0.040).

CVD patients in comparison to controls showed significantly higher systolic BP values (U = 3677, *p* < 0.001) and heart rates (U = 3622, *p* < 0.001). Increased body weight (U = 3034, *p* < 0.001), BMI (U = 2794, *p* < 0.001), waist (U = 2573, *p* < 0.001), hip (U = 3417, *p* < 0.001), and waist/hip ratio (U = 2505, *p* < 0.001) were indicators of the higher metabolic risk. We also registered a higher lifetime cigarette consumption with more pack years (U = 733, *p* = 0.003). The Framingham sum index (U = 2042, *p* < 0.001) and the 10 years risk (U = 2078, *p* < 0.001) were significantly higher in CVD patients. After exact matching for age and sex all statistically significant differences with the exception of systolic BP differences were confirmed.

### 2.2. Inflammation Biomarkers in MDD in Comparison to Healthy Controls

Among inflammatory biomarkers, the general inflammatory marker high sensitivity (hs)CRP, the pro-inflammatory cytokines IL-6 and IL-1β as well as the adhesion molecule sICAM-1 were significantly higher in depressed patients in comparison to controls (Table 2). To rule out possible effects of a divergent age and gender distribution we repeated all comparisons after exact matching for age and sex. All differences remained statistically significant, except for higher sICAM-1 values in MDD patients, which changed to a nonsignificant trend (T = −1.94, *p* = 0.055).

To evaluate putative improvements of cardiovascular risk factors, all inflammatory markers were determined also after remission of depression shortly before discharge of the hospital. Only IL-6 and P selectin declined significantly after treatment with antidepressants (Table 2).

### 2.3. Cardiovascular Risk, Severity of Depression, and Time to Remission in MDD

There was correlative coherence between classical clinical and inflammatory cardiovascular risk factors and the severity of depressive symptoms: Baseline values showed positive correlations with HAM-D17 scores for heart rate (Spearman’s ρ = 0.37, *p* < 0.001), triglycerides (ρ = 0.20, *p* = 0.001), and fasting glucose levels (ρ = 0.25, *p* < 0.001). Weak, but statistically significant negative correlations could be seen for HDL (ρ = −0.15, *p* = 0.02) and for the Framingham sum score (ρ = −0.15, *p* = 0.002) as well as for the 10-years risk score (ρ = −0.15, *p* = 0.003). The current smoking status (numbers of cigarettes) was correlated with hsCRP levels at the time of admission in MDD patients (ρ = 0.59, *p* = 0.045).

Baseline values of inflammation biomarkers revealed weak, but statistically significant positive correlations of HAM-D17 sum scores with hsCRP (ρ = 0.23, *p* < 0.001), sICAM-1 (ρ = 0.35, *p* < 0.001) and IL-1β (ρ = 0.26, *p* = 0.002). IL-6 showed a positive correlation only before discharge (ρ = 0.30, *p* = 0.025).

**Figure 2:**
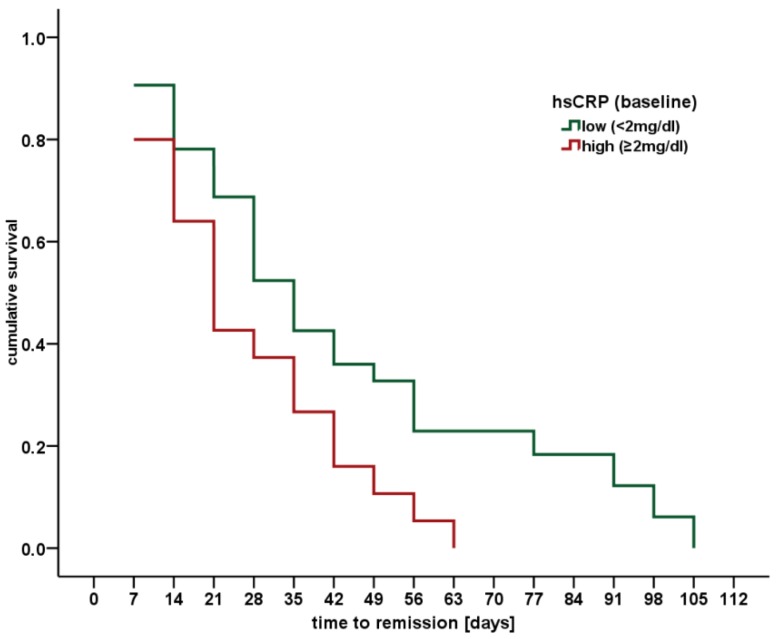
Kaplan-Meyer survival function of time to full remission of MDD for hsCRP status. Estimated likelihood of full remission (HAM-D17 score < 7) based on Mantel-Cox’s regression analysis for hsCRP concentrations at baseline in patients suffering from MDD. Time to full remission was significantly shorter in patients with elevated baseline hsCRP concentrations.

Surprisingly, a specific combination of psychopathology and inflammatory markers seems to affect the time to complete remission (HAM-D17 scores ≤ 7) from MDD: patients more likely to experience a more rapid relief from depressive symptoms suffered from lesser severe MDD (weak positive correlation of HAM-D17 at baseline with time to remission: ρ = 0.28, *p* = 0.045), were younger (ρ = −0.36, *p* < 0.001) and showed a specific inflammatory biomarker profile at baseline. This included higher LDL cholesterol (ρ = 0.39, *p* = 0.037) together with higher IL-1β- (ρ = −0.33, *p* = 0.030) and hsCRP-values (ρ = −0.32, *p* = 0.029) indicating a higher level of chronic inflammation. Also high hsCRP levels >2.0 mg/dL are considered as sign of subclinical inflammation and a biomarker of an enhanced cardiovascular risk [29,30]. Baseline hsCRP values were elevated in 38.5% of our MDD patients. As indicated in Figure 2 the time to reach remission was significantly shorter (mean ± SD high vs. low: 25.6 ± 17.5 vs. 40.0 ± 27.3 days) in patients with elevated baseline CRP (Kaplan-Meyer-analysis, Log Rank (Mantel-Cox) d.f. = 1, *p* = 0.025).

## 3. Discussion

Patients with stable CVD suffered from additional MDD in 15.1% of the cases. Our rate of MDD in CVD patients is in line with other studies investigating the simultaneous presence of both diseases. In the Enhancing Recovery in Coronary Heart Disease (ENRICHD) study CVD patients one month after acute MI, 19.8% suffered from a depressive syndrome and 10.5% fulfilled the criteria for MDD [31]. Also Sørensen et al. found a rate of 10.0% of CVD patients fulfilling ICD-10 criteria for depressive disorders and 7.2% suffered from moderate to severe depression [32]. Our higher incidence rate may be due to the selection of CVD patients suffering from chronic somatic disease, because we recruited the patients not shortly after MI as done in other studies, but in a stable condition during their regular outpatient care in the department of preventive cardiology. An additional reason may be an improved detection of MDD in comparison to other studies due to highly trained psychiatrists on duty in our study. Due to the known elevated inflammation markers in CVD without clear connection to depressive symptoms [33] we omitted the measurement of hsCRP and cytokines in patients suffering from CVD.

Even if depression has been considered as an additional risk factor for CVD independent of classical known risks such as age, gender, vital signs, and blood lipid levels [4,34] the presence of MDD without CVD affects also classical risk factors. In particular, we found a higher number of patients with prehypertensive BP in MDD patients without antihypertensive medication. In spite of antihypertensive treatments, hypertensive states according to JNC-7 criteria [28] were more frequent in CVD. In line with our results, Yan et al. described a higher incidence of hypertension associated with higher depression rating scores in young adults [35].

We detected higher triglycerides and low HDL cholesterol levels in depressed patients in comparison to healthy controls. This profile indicates the considerably enhanced risk for CVD in MDD patients. Differences in fasting glucose levels and waist circumferences as well as a higher rate of smokers in MDD point towards an unhealthy life style (diet, smoking, and low activity) associated with depressive symptoms [36]. Our results suggest that the higher cardiovascular risk in depressive patients is mediated by four of the five component of the metabolic syndrome (triglycerides, fasting glucose, waist circumference, and HDL) rather than by other classical risk factors. Therefore, our findings contribute new data to the current ongoing open debate on the association between depression and metabolic syndrome [37].

Higher inflammatory biomarkers in MDD in comparison to healthy controls support the theory of chronic inflammation and an enhanced cardiovascular risk in MDD. hsCRP represents a stable plasma biomarker for a low-grade systemic inflammation [38]. Elevated IL-6, IL-1β, and sICAM-1 levels in our MDD patients without CVD support the hypothesis of chronic inflammation in MDD. IL-6 stimulates HPA-axis overdrive well known in MDD and CRP production which represents an important risk factor for CVD [39]. This is in line with other studies showing an elevation of inflammatory biomarkers in depression [40] even if underlying mechanisms still are not fully understood and we still don’t know whether the inflammation drives depression or vice versa [41].

In contrast to other studies [26], we could not detect a consistent significant impact of successful antidepressant treatment on all inflammatory markers. Only IL-6 concentrations were significantly lower after treatment. Possible reasons are the divergent influence of antidepressant medication or of MDD itself on differential stages of inflammatory processes, which should be systematically studied in further investigations. We could not detect a reduction of IL-1β concentrations in the peripheral blood. Nevertheless we cannot rule out reduced concentrations after treatment in the brains of our patients which are suggested due to results of preclinical studies: rats exposed to chronic mild stress and treated with the antidepressant fluoxetine for up to 12 days demonstrated lower IL-1β in both plasma and brain [42]. Therefore, these effects should be investigated systematically in further studies.

However, our data indicate that patients remitted from an acute phase of MDD may still have a persistent elevated cardiovascular risk independently from the state of the depressive disorder, because high IL6 indicates an up to three-fold higher risk of sudden cardiac death and high CRP is associated with nonfatal coronary heart disease [43]. This implies a modification of the actually used and possibly insufficient prophylactic strategies preventing CVD in remitted MDD patients.

Moreover, we provide first evidence in our MDD sample that higher hsCRP levels, which indicate subclinical inflammation and higher risks for CVD, are associated with significantly shorter time to remission during antidepressant treatment. This further fosters the discussion about depression influencing immune dysregulation [44] or pathological alterations of the immune system causing depressive symptoms and MDD [26]. In contrast to our results, in the study of Lanquillon et al. in 24 MDD patients higher IL-6 levels predicted worse outcome during the treatment of MDD [45]. However, IL-6 concentrations were not elevated in comparison to healthy controls and elevated CRP was not useful for the prediction of treatment response.

In a recently published study, patients suffering primarily from hypertension and a metabolic syndrome showed also depressive symptoms, but no significant relationship between depression scores and cardiovascular or metabolic risk factors was detected [36]. In our study, smoking showed a positive correlation with hsCRP [46], but we could not confirm correlations with other inflammatory markers. However, correlations of inflammatory biomarkers with severity of depression confirmed a higher cardiovascular risk in more severely depressed patients.

Summarizing the results, our study revealed elevated inflammatory biomarkers in physically completely healthy MDD patients pointing to an enhanced risk for CVD related to the severity of depression. However, a positive impact of inflammation on outcome after antidepressant treatment could be observed. Thus, the immune system’s inflammatory response may not solely facilitate the symptoms of depression [26] but, similar to infection or injury [44], may also represent a constructive consequence of the human body facilitating clinical remission in case of MDD.

Patients suffering from MDD are at specific risk for the development of CVD. For the assessment of this risk the observance of classical cardiovascular risk factors including the Framingham index appears not to be sufficient. Our study suggests that the enhanced cardiovascular risk is mediated additionally by non-traditional risk factors including inflammatory biomarkers and a metabolic syndrome. MDD facilitates an unhealthy life style and consequently favors the development of a metabolic syndrome. On the other hand, biomarkers indicating a chronic inflammation, such as hsCRP, interleukins, and others, suggest that MDD patients are a high risk group for CVD. As a consequence a close monitoring of smoking status, vital signs (blood pressure and heart rate), triglycerides, HDL cholesterol, hsCRP, and, if possible, pro-inflammatory cytokines (IL-1β) and adhesion molecules (sICAM-1) is recommended for cardiovascular risk assessment.

In patients suffering from stable CVD a relatively high rate of MDD could be detected. Due to the fact that MDD in CVD represents a risk factor for subsequent cardiovascular events sufficient treatment of a putative depressive disorder is warranted. Effective antidepressant treatment supports the relief of depression, whereas an improvement of the prognosis and the reduction of cardiovascular mortality could not be proven sufficiently in prospective studies.

For patients suffering from CVD in combination with MDD a cardiac rehabilitation program is recommended, because it was found to increase the levels of physical and mental quality of life and lower also levels of depression [47].

Limitations of our study were the limited case number in comparison to epidemiologic studies. Therefore we were not able to detect small differences in the investigated biomarkers between our three groups. Moreover, up until now, no follow-up investigations to detect longitudinal developments of risk factors and biomarkers and their clinical long-term consequences for the investigated MDD patient group have been performed. Such further investigations are warranted, because they may help to better classify the investigated risk factors and deduce better clinical recommendations.

To meet the requirement of an effective prophylaxis of both cardiovascular disorders in MDD and for diminishing the risk for MDD in CVD, more secondary and primary prevention studies are needed. In MDD early assessment of cardiovascular risk and an early induction of preventive arrangements may be useful. These steps may start with nutritional and life style educations, proceed to dietary arrangements and supplements, and, in case of increased cardiovascular risks, may even include cardio-protective medication. In CVD early diagnosis, treatment and prophylaxis of depression is desirable. This may include psychoeducation, psychosocial therapies, psychotherapeutic approaches, and suitable antidepressant medication.

Even if evidence from randomized controlled trials RCTs still is lacking, in most cases a combination of the mentioned treatments may be of use. From a clinical point of view only the awareness for the described risks, sufficient diagnostics and adequate treatment may help to go against the predicted worldwide rise of the impact of both disorders, CVD and MDD.

## 4. Material and Methods

### 4.1. Study Samples

A total of 333 subjects were recruited within the funding period of four years, 23 patients and controls were excluded due to somatic or psychiatric diagnoses detected after study inclusion or due to withdrawal of consent. Therefore, a total of 310 patients were investigated in the study. Table 1 shows clinical and demographic data for patients and controls.

One hyndred unrelated in-patients suffering from unipolar MDD were recruited. Patients were diagnosed by experienced and trained psychiatrists according to DSM-IV [48] using the Structured Clinical Interview for DSM-IV (SCID). Only patients over 18 years old with an at least moderately severe depressive episode were included. The main inclusion criteria were unipolar depression and a score in the Hamilton Rating Scale for Depression [49] (17-item version, HAM-D17) of at least 17. Exclusion criteria were all other psychiatric comorbidities including e.g., schizophrenia, addiction or mental retardation. Prior to inclusion in the study blood samples were obtained for routine laboratory screening, a medical history was taken and a physical examination was performed by a physician to exclude medical disorders. Clinically relevant medical illness and the concomitant use of antihypertensive medication as well as hormone replacement therapies, alcohol or drug abuse within the last 6 month prior to study inclusion or withdrawal signs led to exclusion from the study. After washout period of at least 3 days prior to the blood sampling for the study patients received non-standardized antidepressant treatments (predominantly Mirtazapine up to 45 mg/day and Venlafaxine up to 300 mg/day) according to clinical requirements, cognitive behavioral treatment and social support. Changes in the depressive state were monitored using the HAM-D17 which was the primary outcome variable, the Montgomery-Åsberg-depression rating scale (MADRS) and the Beck depression inventory (BDI).

One hundred and six outpatients suffering from CVD were recruited from the Department of Internal Medicine—Preventive Cardiology of the Ludwig-Maximilian-University of Munich (sample 2—CVD). Their psychiatric evaluation included also the SCID for DSM-IV diagnoses together with CGI, HAM-D17, MADRS and BDI evaluation. Criteria for inclusion were evidence of CVD documented by confirmed diagnosis of previous myocardial infarction as per hospital discharge summary, or history of CABG or PCI or evidence of ischemic heart disease based on stress electrocardiography confirmed by diagnostic imaging. Patients had to be in stable conditions defined as at least 3 months from an acute episode, intervention or hospitalization for CVD. Thyroid disease, diabetes, hypertension and other chronic conditions had to be well controlled on a stable medical regimen for a minimum of 3 months. Inflammatory biomarkers were not determined in sample 2 due to ongoing treatments with statins.

One hundred and four age- and sex-matched controls of Caucasian ethnicity were recruited at the LMU and screened for psychiatric (SCID) and for medical disorders. A complete medical and social history, a detailed review of systems and complete physical examination were obtained to assess absence of cardiac, cerebral or peripheral vascular disease. Subjects with diabetes were excluded as this is considered a cardiovascular-equivalent disorder. Only healthy individuals negative for both psychiatric and medical disorders entered the study.

### 4.2. Assessment of Vital Signs and Calculation of the Framingham-Index

Height and weight were measured and the body mass index (BMI, kg/m^2^) was calculated. Hip and waist circumference were measured to calculate the hip/waist ratio. At least two blood pressure determinations were made after the patient or control subject had been sitting for at least 5 min with the arm at the heart level. Average values were used for further analysis. The calculation of the Framingham index including the risk factors age, gender, total cholesterol, HDL, systolic and diastolic blood pressure, and smoking status was performed as described elsewhere [50]. It was expressed as a total score and the 10-years risk for CVD incidence.

### 4.3. Biochemical Analyses

#### Inflammatory Risk Factors

All measurements were performed twice from single blinded personal not knowing sample affiliation or clinical details. hsCRP concentrations were determined with a commercially available ELISA (sensitivity 0.1–10 mg/L; DRG Diagnostics, Germany). High sensitivity IL-6 and IL-1β concentrations were measured using ELISA of R&D Systems, Minneapolis according the protocol delivered from the manufacturer. MCP-1, VCAM-1, ICAM-1, E-Selectin, P-Selectin, sCD40-L were determined using ELISAs obtained from IBL (Immuno Biological Lab), Minneapolis, MN, USA according to standard protocols.

### 4.4. Statistical Analysis

All analyses were performed using the Statistical Package for the Social Sciences (SPSS) for Windows (Releases 15-24, SPSS Inc., Chicago, IL, USA and IBM Deutschland GmbH, Ehningen, Germany). The One-Sample Kolmogorov-Smirnov Test was used to test about normal distribution of all variables. In case of a non-normal distribution, the corresponding variables were transformed with the log-transformation to reach a normal distribution before entering parametric testing. In case of persistent deviations from normal distribution non-parametric comparisons of mean values using the Mann-Whitney *U* test for comparison of means were performed. In case of normal distributions mean differences in demographic and clinical variables between patients and controls were compared using univariate analyses of variance (ANOVA procedure) or Student’s *t*-tests in case of continuous variables. In case of categorical variables the frequencies were compared using χ^2^-tests. In addition, we screened cardiovascular risk factors for correlations with predominantly nonparametric variables using Spearman’s rho coefficient. To test for influence of hsCRP status on treatment outcome in MDD patients Kaplan-Meyer survival analysis including Cox-regression was applied.

To rule out significant age and gender effects all comparisons were done after exact matching.

### 4.5. Ethical Approval

The study was approved by the ethics committee of the medical faculty of the Ludwig-Maximilian-University Munich (Project No. 207/03, approval 29 August 2003). Written informed consent was obtained from all patients and control subjects. Patients’ data were anonymized.

## Figures and Tables

**Table 1:** Demographic, clinical, and medical characteristics of samples 1–3 including psychiatric ratings, vital signs, and selected classical cardiovascular risk factors. Vital signs as well as classical cardiovascular risk factors and behavioral variables are indicating an elevated cardiovascular risk in MDD.

	Samples	Sample 1 *	Sample 2	Sample 3 †	Kruskal-Wallis- or χ^2^-Test ‡
Variable		MDD	CVD	Controls	χ^2^, d.f., *p*
*n*		100	106	104	
age (mean ± SD)		46.6 ± 14.8	66.9 ± 7.3	54.7 ± 14.4	100, 2, *p* < 0.001
sex					
(male/female)		37.0%/63.0%	81.1%/18.9%	45.2%/54.8%	46.3, 2, *p* < 0.001 ‡
clinical ratings (baseline)					
CGI-1		5.3 ± 0.5	1.3 ± 0.8	1.0 ± 0.0	205, 2, ***p* < 0.001**
HAM-D17 (mean ± SD)		22.0 ± 5.3	2.4 ± 4.4	0.7 ± 1.2	160, 2, ***p* < 0.001**
MADRS (mean ± SD)		31.8 ± 7.4	3.2 ± 6.0	0.7 ± 1.3	162, 2, ***p* < 0.001**
BDI (mean ± SD)		26.4 ± 9.1	7.0 ± 5.1	2.8 ± 3.2	130, 2, ***p* < 0.001**
Vital signs and classical cardiovascular risk factors					
blood pressure (systolic)		129.2 ± 13.5	138.6 ± 19.1	128.6 ± 19.1	18.7, 2, ***p* < 0.001**
blood pressure (diastolic)		79.7 ± 8.2	78.3 ± 10.9	76.8 ± 11.9	4.63, 2, n.s. ^§^
heart rate (beats/minute)		85.7 ± 14.7	63.8 ± 10.7	68.9 ± 10.1	104, 2, ***p* < 0.001**
total cholesterol (mg/dL)		206.2 ± 58.1	n.d.	223.8 ± 48.1	4.15, 1, *p* = 0.042
LDL (mg/dL)		125.2 ± 42.8	n.d.	136.9 ± 45.6	2.30, 1, n.s.
triglycerides (mg/dL)		148.8 ± 100.3	n.d.	100.6 ± 51.6	10.5, 1, ***p* = 0.001**
HDL (mg/dL)		58.4 ± 17.1	n.d.	66.1 ± 19.8	5.67, 1, ***p* = 0.017**
fasting glucose (mg/dL)		95.1 ± 18.2	n.d.	86.9 ± 11.6	5.41, 1, ***p* = 0.02**
body weight (kg)		72.9 ± 13.6	82.8 ± 14.7	72.3 ± 11.8	32.9, 2, *p* < 0.001
body mass index (kg/m^2^)		25.3 ± 4.0	28.0 ± 4.4	24.4 ± 2.9	35.8, 2, ***p* < 0.001**
waist circumference (cm)		96.9 ± 12.3	101.9 ± 12.9	90.1 ± 12.1	34.2, 2, ***p* < 0.001**
hip circumference (cm)		105.3 ± 10.7	105.8 ± 9.5	100.6 ± 8.4	12.5, 2, *p* = 0.002
waist-hip-ratio		0.92 ± 0.12	0.96 ± 0.07	0.90 ± 0.09	35.9, 2, *p* < 0.001
smoker/non-smoker (%)		42.9%/57.1%	9.0%/91.0%	14.6%/85.4%	34.1, 2, ***p* < 0.001 ‡**
pack years (20 cigarettes/day * years)		18.8 ± 13.4	35.7 ± 30.7	18.8 ± 20.3	9.96, 2, *p* = 0.007
Framingham-index (total)		2.77 ± 5.74	9.69 ± 2.69	4.09 ± 5.86	99.3, 2, *p* < 0.001
Framingham-index, 10 years-risk (%)		4.94 ± 5.62	11.47 ± 7.11	5.85 ± 5.78	96.4, 2, *p* < 0.001

dropouts: *n* = 16; MDD = major depressive disorder; CVD = cardiovascular disorder; ANOVA = univariate analysis of variance; d.f. = degrees of freedom; SD = standard deviation; n.s. = not statistically significant; n.d. = not done (due to treatment with statins); CGI-1 = Clinical global impression scale, Item 1—severity of disease; HAM-D17 = Hamilton rating scale for depression, 17-item version; MADRS = depression rating scale; BDI = Beck depression inventory; LDL = low density lipoproteins; HDL = high density lipoproteins; * medical comorbidities were exclusion criteria for sample 1; † medical and psychiatric comorbidities were exclusion criteria for sample 3; bold = statistical significant differences between MDD and controls confirmed (Mann-Whitney-U test) after exact age and sex matching; ‡ χ^2^-test in case of categorical variables (continuous variables were evaluated by univariate analysis of variance); ^§^ Man-Whitney-U test: *p* = 0.005.

**Table 2:** Inflammation biomarkers and risk factors.

	Samples	Sample 1		*t*-Test *	Sample 3	*t*-Test †
		MDD			Controls	
Variable		Baseline	Discharge	T, *p*	Baseline	T, *p*
inflammation marker						
hsCRP (mg/L)		3.07 ± 3.7	3.97 ± 4.4	0.23, n.s.	1.37 ± 1.2	**4.25, *p* < 0.001**
pro-inflammatory cytokines						
interleukin 1β (IL-1 β) (pg/mL)		1.08 ± 1.1	1.34 ± 1.0	0.88, n.s.	0.54 ± 0.6	**4.10, *p* < 0.001**
interleukin 6 (IL-6) (pg/mL)		1.58 ± 1.5	1.35 ± 1.7	2.26, *p* = 0.027	1.32 ± 1.3	**2.64, *p* = 0.009**
adhesion molecules						
P selectin (ng/mL)		150.4 ± 102.4	114.4 ± 78.9	2.56, *p* = 0.013	184.2 ± 146.0	−1.47, n.s.
E selectin (ng/mL)		54.6 ± 29.0	58.0 ± 32.0	−0.69, n.s.	46.5 ± 27.2	1.76, n.s.
MCP-1 (pg/mL)		221.3 ± 149.7	301.8 ± 179.2	−2.92, *p* = 0.005	256.5 ± 140.8	−1.54, n.s.
sICAM-1 (ng/mL)		535.4 ± 210.0	555.0 ± 209.5	−0.92, n.s.	360.6 ± 107.7	**5.90, *p* < 0.001**
sVCAM-1 (ng/mL)		486.1 ± 182.0	533.2 ± 221.4	−1.62, n.s.	552.3 ± 142.2	**−4.41, *p* = 0.017**
costimulatory glycoprotein						
sCD40 (ng/mL)		10.4 ± 3.7	11.3 ± 3.7	−2.42, *p* = 0.019	10.1 ± 4.2	0.59, n.s.

Inflammation biomarkers in MDD patients. Comparison to healthy controls: hsCRP, IL-1β, and sICAM-1 were significantly elevated in comparison to healthy controls. Other markers showed only nonsignificant trends or were even lower in MDD. Comparison before and after treatment of depression (at baseline and before discharge of the hospital): Reduced IL-6 and P selectin after antidepressant treatment. * Student’s *t*-test for dependent samples: sample 1 baseline vs. discharge; † Student’s *t*-test for independent samples: baseline sample 1 vs. baseline sample 3; n.s. = not statistically significant; bold = statistical significant differences between MDD and controls confirmed after exact age and sex matching (*t*-test and Mann-Whitney-U test).
